# Supplementary figures and images for: Seed germination and early seedling survival of the invasive species Prosopis juliflora (Fabaceae) depend on habitat and seed dispersal mode in the Caatinga dry forest
Source: PeerJ. 2020 Sep 3;8:e9607. doi: 10.7717/peerj.9607 (PMC7474883; doi:10.7717/peerj.9607)

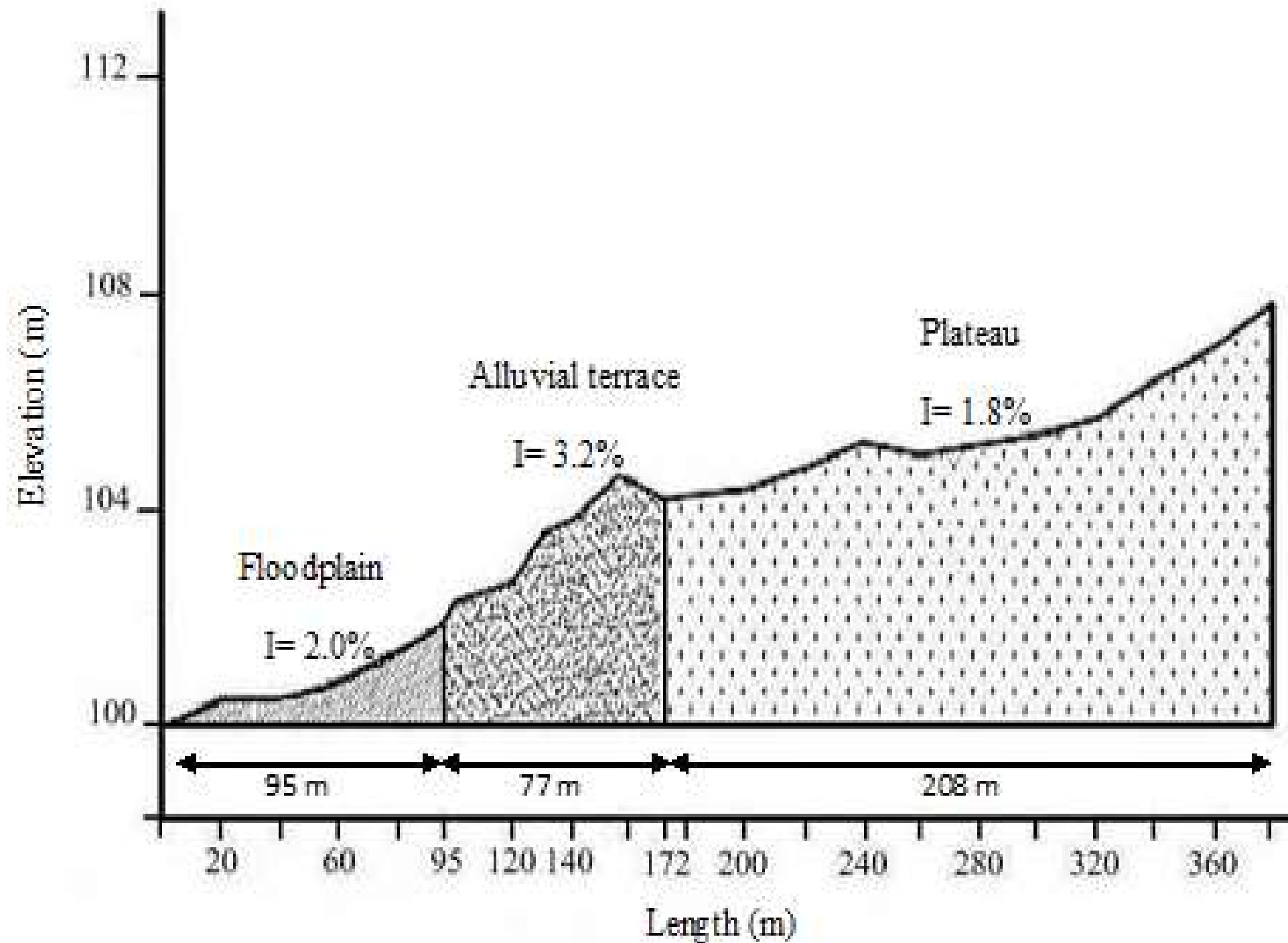

Supplement: Supplemental Information 1 — I= inclination. [file peerj-08-9607-s001.pdf]

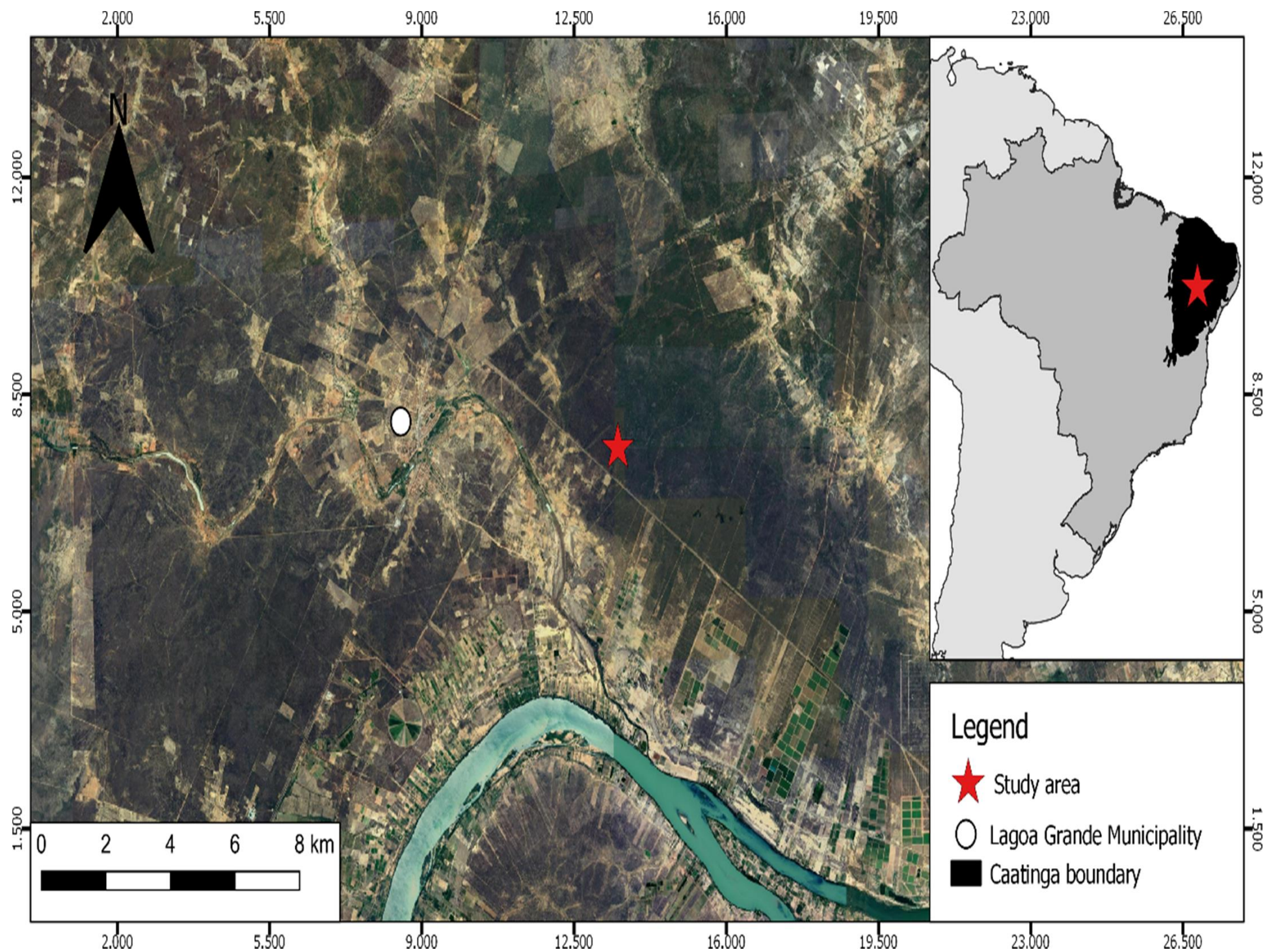

Supplement: Supplemental Information 2 — Google (2020). Map data ©2015 Google. Retrieved with QuickMapServices plugin for QGIS 3.4.14. [file peerj-08-9607-s002.pdf]
