# Supplementary material for: Seed germination and early seedling survival of the invasive species Prosopis juliflora (Fabaceae) depend on habitat and seed dispersal mode in the Caatinga dry forest
Source: PeerJ. 2020 Sep 3;8:e9607. doi: 10.7717/peerj.9607 (PMC7474883; doi:10.7717/peerj.9607)
Supplement: Supplemental Information 3 [file peerj-08-9607-s003.doc]

| *x* | *L_x_* | *d_x_* | *S_x_* | *lx* | *e_x_* | *Q_x_* | *E_x_* | *T_x_* |  | *L_x_* | *d_x_* | *S_x_* | *lx* | *e_x_* | *Q_x_* | *E_x_* | *T_x_* | DS |
| --- | --- | --- | --- | --- | --- | --- | --- | --- | --- | --- | --- | --- | --- | --- | --- | --- | --- | --- |
|  | Surface | | | | | | | |  | Buried | | | | | | | |  |
| 0 | 13 | 6 | 0.54 | 1.00 | 4.46 | 0.46 | 10.0 | 58.0 |  | 474 | 438 | 0.08 | 1.00 | 0.04 | 0.92 | 0.5 | 20.3 | G |
| 15 | 7 | 0 | 1.00 | 0.54 | 6.86 | 0.00 | 7.0 | 48.0 |  | 36 | 4 | 0.89 | 0.08 | 0.55 | 0.11 | 0.7 | 19.8 |  |
| 30 | 7 | 1 | 0.86 | 0.54 | 5.86 | 0.14 | 6.5 | 41.0 |  | 32 | 14 | 0.56 | 0.07 | 0.60 | 0.44 | 0.5 | 19.1 | S |
| 45 | 6 | 3 | 0.50 | 0.46 | 5.75 | 0.50 | 4.5 | 34.5 |  | 18 | 9 | 0.50 | 0.04 | 1.03 | 0.50 | 0.7 | 18.5 |  |
| 60 | 3 | 1 | 0.67 | 0.23 | 10.00 | 0.33 | 2.5 | 30.0 |  | 9 | 1 | 0.89 | 0.02 | 1.98 | 0.11 | 0.9 | 17.8 |  |
| 75 | 2 | 0 | 1.00 | 0.15 | 13.75 | 0.00 | 2.0 | 27.5 |  | 8 | 1 | 0.88 | 0.02 | 2.12 | 0.13 | 0.9 | 17.0 |  |
| 90 | 2 | 0 | 1.00 | 0.15 | 12.75 | 0.00 | 2.0 | 25.5 |  | 7 | 0 | 1.00 | 0.02 | 2.29 | 0.00 | 1.0 | 16.0 |  |
| 105 | 2 | 0 | 1.00 | 0.15 | 11.75 | 0.00 | 2.0 | 23.5 |  | 7 | 0 | 1.00 | 0.02 | 2.15 | 0.00 | 1.0 | 15.0 |  |
| 120 | 2 | 0 | 1.00 | 0.15 | 10.75 | 0.00 | 2.0 | 21.5 |  | 7 | 0 | 1.00 | 0.02 | 2.00 | 0.00 | 0.9 | 14.0 |  |
| 135 | 2 | 0 | 1.00 | 0.15 | 9.75 | 0.00 | 2.0 | 19.5 |  | 7 | 1 | 0.86 | 0.02 | 1.87 | 0.14 | 0.8 | 13.1 |  |
| 150 | 2 | 0 | 1.00 | 0.15 | 8.75 | 0.00 | 2.0 | 17.5 |  | 6 | 2 | 0.67 | 0.01 | 2.06 | 0.33 | 0.6 | 12.3 |  |
| 165 | 2 | 0 | 1.00 | 0.15 | 7.75 | 0.00 | 2.0 | 15.5 |  | 4 | 2 | 0.50 | 0.01 | 2.94 | 0.50 | 0.8 | 11.8 |  |
| 180 | 2 | 0 | 1.00 | 0.15 | 6.75 | 0.00 | 2.0 | 13.5 |  | 2 | 0 | 1.00 | 0.00 | 5.50 | 0.00 | 1.0 | 11.0 |  |
| 195 | 2 | 1 | 0.50 | 0.15 | 5.75 | 0.50 | 1.5 | 11.5 |  | 2 | 0 | 1.00 | 0.00 | 5.00 | 0.00 | 1.0 | 10.0 |  |
| 210 | 1 | 0 | 1.00 | 0.08 | 10.00 | 0.00 | 1.0 | 10.0 |  | 2 | 0 | 1.00 | 0.00 | 4.50 | 0.00 | 0.8 | 9.0 |  |
| 225 | 1 | 0 | 1.00 | 0.08 | 9.00 | 0.00 | 1.0 | 9.0 |  | 2 | 1 | 0.50 | 0.00 | 4.13 | 0.50 | 0.8 | 8.3 |  |
| 240 | 1 | 0 | 1.00 | 0.08 | 8.00 | 0.00 | 1.0 | 8.0 |  | 1 | 0 | 1.00 | 0.00 | 7.50 | 0.00 | 1.0 | 7.5 |  |
| 255 | 1 | 0 | 1.00 | 0.08 | 7.00 | 0.00 | 1.0 | 7.0 |  | 1 | 0 | 1.00 | 0.00 | 6.50 | 0.00 | 1.0 | 6.5 |  |
| 270 | 1 | 0 | 1.00 | 0.08 | 6.00 | 0.00 | 1.0 | 6.0 |  | 1 | 0 | 1.00 | 0.00 | 5.50 | 0.00 | 1.0 | 5.5 |  |
| 285 | 1 | 0 | 1.00 | 0.08 | 5.00 | 0.00 | 1.0 | 5.0 |  | 1 | 0 | 1.00 | 0.00 | 4.50 | 0.00 | 1.0 | 4.5 |  |
| 300 | 1 | 0 | 1.00 | 0.08 | 4.00 | 0.00 | 1.0 | 4.0 |  | 1 | 0 | 1.00 | 0.00 | 3.50 | 0.00 | 1.0 | 3.5 |  |
| 315 | 1 | 0 | 1.00 | 0.08 | 3.00 | 0.00 | 1.0 | 3.0 |  | 1 | 0 | 1.00 | 0.00 | 2.50 | 0.00 | 1.0 | 2.5 |  |
| 330 | 1 | 0 | 1.00 | 0.08 | 2.00 | 0.00 | 1.0 | 2.0 |  | 1 | 0 | 1.00 | 0.00 | 1.50 | 0.00 | 1.0 | 1.5 |  |
| 345 | 1 | 0 | 1.00 | 0.08 | 1.00 | 0.00 | 1.0 | 1.0 |  | 1 | 0 | 1.00 | 0.00 | 0.50 | 0.00 | 0.5 | 0.5 |  |
|  | Cattle manure | | | | | | | |  | Mule manure | | | | | | | |  |
| 0 | 205 | 89 | 0.57 | 1.00 | 2.67 | 0.43 | 160.5 | 546.5 |  | 134 | 54 | 0.60 | 1.00 | 2.91 | 0.40 | 107.0 | 390.5 | G |
| 15 | 116 | 25 | 0.78 | 0.57 | 3.33 | 0.22 | 103.5 | 386.0 |  | 80 | 16 | 0.80 | 0.60 | 3.54 | 0.20 | 72.0 | 283.5 |  |
| 30 | 91 | 51 | 0.44 | 0.44 | 3.10 | 0.56 | 65.5 | 282.5 |  | 64 | 38 | 0.41 | 0.48 | 3.30 | 0.59 | 45.0 | 211.5 | S |
| 45 | 40 | 19 | 0.53 | 0.20 | 5.43 | 0.48 | 30.5 | 217.0 |  | 26 | 4 | 0.85 | 0.19 | 6.40 | 0.15 | 24.0 | 166.5 |  |
| 60 | 21 | 1 | 0.95 | 0.10 | 8.88 | 0.05 | 20.5 | 186.5 |  | 22 | 6 | 0.73 | 0.16 | 6.48 | 0.27 | 19.0 | 142.5 |  |
| 75 | 20 | 1 | 0.95 | 0.10 | 8.30 | 0.05 | 19.5 | 166.0 |  | 16 | 0 | 1.00 | 0.12 | 7.72 | 0.00 | 16.0 | 123.5 |  |
| 90 | 19 | 0 | 1.00 | 0.09 | 7.71 | 0.00 | 19.0 | 146.5 |  | 16 | 1 | 0.94 | 0.12 | 6.72 | 0.06 | 15.5 | 107.5 |  |
| 105 | 19 | 0 | 1.00 | 0.09 | 6.71 | 0.00 | 19.0 | 127.5 |  | 15 | 0 | 1.00 | 0.11 | 6.13 | 0.00 | 15.0 | 92.0 |  |
| 120 | 19 | 3 | 0.84 | 0.09 | 5.71 | 0.16 | 17.5 | 108.5 |  | 15 | 1 | 0.93 | 0.11 | 5.13 | 0.07 | 14.5 | 77.0 |  |
| 135 | 16 | 2 | 0.88 | 0.08 | 5.69 | 0.13 | 15.0 | 91.0 |  | 14 | 0 | 1.00 | 0.10 | 4.46 | 0.00 | 14.0 | 62.5 |  |
| 150 | 14 | 6 | 0.57 | 0.07 | 5.43 | 0.43 | 11.0 | 76.0 |  | 14 | 3 | 0.79 | 0.10 | 3.46 | 0.21 | 12.5 | 48.5 |  |
| 165 | 8 | 1 | 0.88 | 0.04 | 8.13 | 0.13 | 7.5 | 65.0 |  | 11 | 1 | 0.91 | 0.08 | 3.27 | 0.09 | 10.5 | 36.0 |  |
| 180 | 7 | 1 | 0.86 | 0.03 | 8.21 | 0.14 | 6.5 | 57.5 |  | 10 | 4 | 0.60 | 0.07 | 2.55 | 0.40 | 8.0 | 25.5 |  |
| 195 | 6 | 0 | 1.00 | 0.03 | 8.50 | 0.00 | 6.0 | 51.0 |  | 6 | 3 | 0.50 | 0.04 | 2.92 | 0.50 | 4.5 | 17.5 |  |
| 210 | 6 | 0 | 1.00 | 0.03 | 7.50 | 0.00 | 6.0 | 45.0 |  | 3 | 1 | 0.67 | 0.02 | 4.33 | 0.33 | 2.5 | 13.0 |  |
| 225 | 6 | 1 | 0.83 | 0.03 | 6.50 | 0.17 | 5.5 | 39.0 |  | 2 | 0 | 1.00 | 0.01 | 5.25 | 0.00 | 2.0 | 10.5 |  |
| 240 | 5 | 0 | 1.00 | 0.02 | 6.70 | 0.00 | 5.0 | 33.5 |  | 2 | 1 | 0.50 | 0.01 | 4.25 | 0.50 | 1.5 | 8.5 |  |
| 255 | 5 | 1 | 0.80 | 0.02 | 5.70 | 0.20 | 4.5 | 28.5 |  | 1 | 0 | 1.00 | 0.01 | 7.00 | 0.00 | 1.0 | 7.0 |  |
| 270 | 4 | 0 | 1.00 | 0.02 | 6.00 | 0.00 | 4.0 | 24.0 |  | 1 | 0 | 1.00 | 0.01 | 6.00 | 0.00 | 1.0 | 6.0 |  |
| 285 | 4 | 0 | 1.00 | 0.02 | 5.00 | 0.00 | 4.0 | 20.0 |  | 1 | 0 | 1.00 | 0.01 | 5.00 | 0.00 | 1.0 | 5.0 |  |
| 300 | 4 | 0 | 1.00 | 0.02 | 4.00 | 0.00 | 4.0 | 16.0 |  | 1 | 0 | 1.00 | 0.01 | 4.00 | 0.00 | 1.0 | 4.0 |  |
| 315 | 4 | 0 | 1.00 | 0.02 | 3.00 | 0.00 | 4.0 | 12.0 |  | 1 | 0 | 1.00 | 0.01 | 3.00 | 0.00 | 1.0 | 3.0 |  |
| 330 | 4 | 0 | 1.00 | 0.02 | 2.00 | 0.00 | 4.0 | 8.0 |  | 1 | 0 | 1.00 | 0.01 | 2.00 | 0.00 | 1.0 | 2.0 |  |
| 345 | 4 | 0 | 1.00 | 0.02 | 1.00 | 0.00 | 4.0 | 4.0 |  | 1 | 0 | 1.00 | 0.01 | 1.00 | 0.00 | 1.0 | 1.0 |  |

*x*= age interval (days), *L_x_*= number of live individuals at the beginning of age *x*, *d_x_*= number of individuals dead during each age interval, *S_x_*= percentage of individuals alive per age interval *x*, *lx*= age-specific survival rate, *e_x_*= life expectancy for individuals of age *x*, *q_x_*= mortality rate per age interval, *E_x_*= age structure and *T_x_*= total number of individuals of age beyond that age. DS= developmental stage, G= germination and S= seedling.
