# Supplementary material for: Seed germination and early seedling survival of the invasive species Prosopis juliflora (Fabaceae) depend on habitat and seed dispersal mode in the Caatinga dry forest
Source: PeerJ. 2020 Sep 3;8:e9607. doi: 10.7717/peerj.9607 (PMC7474883; doi:10.7717/peerj.9607)
Supplement: Supplemental Information 4 [file peerj-08-9607-s004.doc]

| *x* | *L_x_* | *d_x_* | *S_x_* | *lx* | *e_x_* | *Q_x_* | *E_x_* | *T_x_* |  | *L_x_* | *d_x_* | *S_x_* | *lx* | *e_x_* | *Q_x_* | *E_x_* | *T_x_* | DS |
| --- | --- | --- | --- | --- | --- | --- | --- | --- | --- | --- | --- | --- | --- | --- | --- | --- | --- | --- |
|  | Surface | | | | | | | |  | Buried | | | | | | | |  |
| 0 | 53 | 31 | 0.42 | 1.00 | 2.10 | 0.58 | 37.5 | 111.5 |  | 334 | 240 | 0.28 | 1.00 | 1.33 | 0.72 | 214.0 | 445.5 | G |
| 15 | 22 | 4 | 0.82 | 0.42 | 3.36 | 0.18 | 20.0 | 74.0 |  | 94 | 15 | 0.84 | 0.28 | 2.46 | 0.16 | 86.5 | 231.5 |  |
| 30 | 18 | 4 | 0.78 | 0.34 | 3.00 | 0.22 | 16.0 | 54.0 |  | 79 | 44 | 0.44 | 0.24 | 1.84 | 0.56 | 57.0 | 145.0 | S |
| 45 | 14 | 5 | 0.64 | 0.26 | 2.71 | 0.36 | 11.5 | 38.0 |  | 35 | 15 | 0.57 | 0.11 | 2.51 | 0.43 | 27.5 | 88.0 |  |
| 60 | 9 | 3 | 0.67 | 0.17 | 2.94 | 0.33 | 7.5 | 26.5 |  | 20 | 5 | 0.75 | 0.06 | 3.03 | 0.25 | 17.5 | 60.5 |  |
| 75 | 6 | 2 | 0.67 | 0.11 | 3.17 | 0.33 | 5.0 | 19.0 |  | 15 | 4 | 0.73 | 0.05 | 2.87 | 0.27 | 13.0 | 43.0 |  |
| 90 | 4 | 0 | 1.00 | 0.08 | 3.50 | 0.00 | 4.0 | 14.0 |  | 11 | 2 | 0.82 | 0.03 | 2.73 | 0.18 | 10.0 | 30.0 |  |
| 105 | 4 | 1 | 0.75 | 0.08 | 2.50 | 0.25 | 3.5 | 10.0 |  | 9 | 1 | 0.89 | 0.03 | 2.22 | 0.11 | 8.5 | 20.0 |  |
| 120 | 3 | 1 | 0.67 | 0.06 | 2.17 | 0.33 | 2.5 | 6.5 |  | 8 | 3 | 0.63 | 0.02 | 1.44 | 0.38 | 6.5 | 11.5 |  |
| 135 | 2 | 0 | 1.00 | 0.04 | 2.00 | 0.00 | 2.0 | 4.0 |  | 5 | 4 | 0.20 | 0.02 | 1.00 | 0.80 | 3.0 | 5.0 |  |
| 150 | 2 | 0 | 1.00 | 0.04 | 1.00 | 0.00 | 2.0 | 2.0 |  | 1 | 0 | 1.00 | 0.00 | 2.00 | 0.00 | 1.0 | 2.0 |  |
| 165 | - | - | - | - | - | - | - | - |  | 1 | 0 | 1.00 | 0.00 | 1.00 | 0.00 | 1.0 | 1.0 |  |
|  | Cattle manure | | | | | | | |  | Mule manure | | | | | | | |  |
| 0 | 172 | 43 | 0.75 | 1.00 | 2.61 | 0.25 | 150.5 | 449.5 |  | 117 | 26 | 0.78 | 1.00 | 2.45 | 0.22 | 104.0 | 286.5 | G |
| 15 | 129 | 12 | 0.91 | 0.75 | 2.32 | 0.09 | 123.0 | 299.0 |  | 91 | 15 | 0.84 | 0.78 | 2.01 | 0.16 | 83.5 | 182.5 |  |
| 30 | 117 | 55 | 0.53 | 0.68 | 1.50 | 0.47 | 89.5 | 176.0 |  | 76 | 44 | 0.42 | 0.65 | 1.30 | 0.58 | 54.0 | 99.0 | S |
| 45 | 62 | 44 | 0.29 | 0.36 | 1.40 | 0.71 | 40.0 | 86.5 |  | 32 | 25 | 0.22 | 0.27 | 1.41 | 0.78 | 19.5 | 45.0 |  |
| 60 | 18 | 4 | 0.78 | 0.11 | 2.58 | 0.22 | 16.0 | 46.5 |  | 7 | 2 | 0.71 | 0.06 | 3.64 | 0.29 | 6.0 | 25.5 |  |
| 75 | 14 | 4 | 0.71 | 0.08 | 2.18 | 0.29 | 12.0 | 30.5 |  | 5 | 1 | 0.80 | 0.04 | 3.90 | 0.20 | 4.5 | 19.5 |  |
| 90 | 10 | 1 | 0.90 | 0.06 | 1.85 | 0.10 | 9.5 | 18.5 |  | 4 | 0 | 1.00 | 0.03 | 3.75 | 0.00 | 4.0 | 15.0 |  |
| 105 | 9 | 7 | 0.22 | 0.05 | 1.00 | 0.78 | 5.5 | 9.0 |  | 4 | 0 | 1.00 | 0.03 | 2.75 | 0.00 | 4.0 | 11.0 |  |
| 120 | 2 | 1 | 0.50 | 0.01 | 1.75 | 0.50 | 1.5 | 3.5 |  | 4 | 2 | 0.50 | 0.03 | 1.75 | 0.50 | 3.0 | 7.0 |  |
| 135 | 1 | 0 | 1.00 | 0.01 | 2.00 | 0.00 | 1.0 | 2.0 |  | 2 | 0 | 1.00 | 0.02 | 2.00 | 0.00 | 2.0 | 4.0 |  |
| 150 | 1 | 0 | 1.00 | 0.01 | 1.00 | 0.00 | 1.0 | 1.0 |  | 2 | 0 | 1.00 | 0.02 | 1.00 | 0.00 | 2.0 | 2.0 |  |

*x*= age interval (days), *L_x_*= number of live individuals at the beginning of age *x*, *d_x_*= number of individuals dead during each age interval, *S_x_*= percentage of individuals alive per age interval *x*, *lx*= age-specific survival rate, *e_x_*= life expectancy for individuals of age *x*, *q_x_*= mortality rate per age interval, *E_x_*= age structure and *T_x_*= total number of individuals of age beyond that age. DS= developmental stage, G= germination and S= seedling.
