# Supplementary material for: Seed germination and early seedling survival of the invasive species Prosopis juliflora (Fabaceae) depend on habitat and seed dispersal mode in the Caatinga dry forest
Source: PeerJ. 2020 Sep 3;8:e9607. doi: 10.7717/peerj.9607 (PMC7474883; doi:10.7717/peerj.9607)
Supplement: Supplemental Information 5 [file peerj-08-9607-s005.doc]

| *x* | *L_x_* | *d_x_* | | *S_x_* | *lx* | *e_x_* | *Q_x_* | | *E_x_* | *T_x_* |  | *L_x_* | *d_x_* | *S_x_* | *lx* | *e_x_* | *Q_x_* | *E_x_* | *T_x_* | DS |
| --- | --- | --- | --- | --- | --- | --- | --- | --- | --- | --- | --- | --- | --- | --- | --- | --- | --- | --- | --- | --- |
|  | Surface | | | | | | | | | |  | Buried | | | | | | | |  |
| 0 | 7 | | 6 | 0.14 | 1.00 | 1.00 | | 0.86 | 4.0 | 7.0 |  | 16 | 15 | 0.06 | 1.00 | 1.03 | 0.94 | 8.5 | 16.5 | G |
| 15 | 1 | | 0 | 1.00 | 0.14 | 3.00 | | 0.00 | 1.0 | 3.0 |  | 1 | 0 | 1.00 | 0.06 | 8.00 | 0.00 | 1.0 | 8.0 |  |
| 30 | 1 | | 0 | 1.00 | 0.14 | 2.00 | | 0.00 | 1.0 | 2.0 |  | 1 | 0 | 1.00 | 0.06 | 7.00 | 0.00 | 1.0 | 7.0 | S |
| 45 | 1 | | 0 | 1.00 | 0.14 | 1.00 | | 0.00 | 1.0 | 1.0 |  | 1 | 0 | 1.00 | 0.06 | 6.00 | 0.00 | 1.0 | 6.0 |  |
| 60 | - | | - | - | - | - | | - | - | - |  | 1 | 0 | 1.00 | 0.06 | 5.00 | 0.00 | 1.0 | 5.0 |  |
| 75 | - | | - | - | - | - | | - | - | - |  | 1 | 0 | 1.00 | 0.06 | 4.00 | 0.00 | 1.0 | 4.0 |  |
| 90 | - | | - | - | - | - | | - | - | - |  | 1 | 0 | 1.00 | 0.06 | 3.00 | 0.00 | 1.0 | 3.0 |  |
| 105 | - | | - | - | - | - | | - | - | - |  | 1 | 0 | 1.00 | 0.06 | 2.00 | 0.00 | 1.0 | 2.0 |  |
| 120 | - | | - | - | - | - | | - | - | - |  | 1 | 0 | 1.00 | 0.06 | 1.00 | 0.00 | 1.0 | 1.0 |  |
|  | Cattle manure | | | | | | | | | |  | Mule manure | | | | | | | |  |
| 0 | 129 | | 112 | 0.13 | 1.00 | 1.14 | | 0.87 | 73.0 | 147.0 |  | 0 | 92 | 87 | 0.05 | 1.00 | 0.95 | 48.5 | 53.5 | G |
| 15 | 17 | | 1 | 0.94 | 0.13 | 4.35 | | 0.06 | 16.5 | 74.0 |  | 15 | 5 | 4 | 0.20 | 0.05 | 0.80 | 3.0 | 5.0 |  |
| 30 | 16 | | 6 | 0.63 | 0.12 | 3.59 | | 0.38 | 13.0 | 57.5 |  | 30 | 1 | 0 | 1.00 | 0.01 | 0.00 | 1.0 | 2.0 | S |
| 45 | 10 | | 3 | 0.70 | 0.08 | 4.45 | | 0.30 | 8.5 | 44.5 |  | 45 | 1 | 0 | 1.00 | 0.01 | 0.00 | 1.0 | 1.0 |  |
| 60 | 7 | | 0 | 1.00 | 0.05 | 5.14 | | 0.00 | 7.0 | 36.0 |  | - | - | - | - | - | - | - | - |  |
| 75 | 7 | | 0 | 1.00 | 0.05 | 4.14 | | 0.00 | 7.0 | 29.0 |  | - | - | - | - | - | - | - | - |  |
| 90 | 7 | | 3 | 0.57 | 0.05 | 3.14 | | 0.43 | 5.5 | 22.0 |  | - | - | - | - | - | - | - | - |  |
| 105 | 4 | | 0 | 1.00 | 0.03 | 4.13 | | 0.00 | 4.0 | 16.5 |  | - | - | - | - | - | - | - | - |  |
| 120 | 4 | | 1 | 0.75 | 0.03 | 3.13 | | 0.25 | 3.5 | 12.5 |  | - | - | - | - | - | - | - | - |  |
| 135 | 3 | | 0 | 1.00 | 0.02 | 1.00 | | 0.00 | 3.0 | 3.0 |  | - | - | - | - | - | - | - | - |  |
| 150 | 3 | | 0 | 1.00 | 0.02 | 2.00 | | 0.00 | 3.0 | 6.0 |  | - | - | - | - | - | - | - | - |  |
| 165 | 3 | | 0 | 1.00 | 0.02 | 1.00 | | 0.00 | 3.0 | 3.0 |  | - | - | - | - | - | - | - | - |  |

*x*= age interval (days), *L_x_*= number of live individuals at the beginning of age *x*, *d_x_*= number of individuals dead during each age interval, *S_x_*= percentage of individuals alive per age interval *x*, *lx*= age-specific survival rate, *e_x_*= life expectancy for individuals of age *x*, *q_x_*= mortality rate per age interval, *E_x_*= age structure and *T_x_*= total number of individuals of age beyond that age. DS= developmental stage, G= germination and S= seedling.
