# Supplementary material for: Seed germination and early seedling survival of the invasive species Prosopis juliflora (Fabaceae) depend on habitat and seed dispersal mode in the Caatinga dry forest
Source: PeerJ. 2020 Sep 3;8:e9607. doi: 10.7717/peerj.9607 (PMC7474883; doi:10.7717/peerj.9607)
Supplement: Supplemental Information 6 — Habitats: flooding plain (1), terrace (2) and plateau (3). Seed dispersal mode: on the ground (1), buried (2), cattle-dispersed seeds (3), and mule-dispersed seeds (4). [file peerj-08-9607-s006.docx]

| Gerninated seeds | Sample | Habitat | Dispersal mode |
| --- | --- | --- | --- |
| 1 | 1 | 1 | 1 |
| 0 | 2 | 1 | 1 |
| 9 | 3 | 1 | 1 |
| 0 | 4 | 1 | 1 |
| 0 | 5 | 1 | 1 |
| 1 | 6 | 1 | 1 |
| 1 | 7 | 1 | 1 |
| 0 | 8 | 1 | 1 |
| 1 | 9 | 1 | 1 |
| 0 | 10 | 1 | 1 |
| 17 | 1 | 2 | 1 |
| 6 | 2 | 2 | 1 |
| 1 | 3 | 2 | 1 |
| 17 | 4 | 2 | 1 |
| 2 | 5 | 2 | 1 |
| 8 | 6 | 2 | 1 |
| 0 | 7 | 2 | 1 |
| 0 | 8 | 2 | 1 |
| 0 | 9 | 2 | 1 |
| 2 | 10 | 2 | 1 |
| 0 | 1 | 3 | 1 |
| 5 | 2 | 3 | 1 |
| 0 | 3 | 3 | 1 |
| 0 | 4 | 3 | 1 |
| 0 | 5 | 3 | 1 |
| 0 | 6 | 3 | 1 |
| 0 | 7 | 3 | 1 |
| 2 | 8 | 3 | 1 |
| 0 | 9 | 3 | 1 |
| 0 | 10 | 3 | 1 |
| 44 | 1 | 1 | 2 |
| 45 | 2 | 1 | 2 |
| 41 | 3 | 1 | 2 |
| 73 | 4 | 1 | 2 |
| 66 | 5 | 1 | 2 |
| 5 | 6 | 1 | 2 |
| 42 | 7 | 1 | 2 |
| 80 | 8 | 1 | 2 |
| 68 | 9 | 1 | 2 |
| 10 | 10 | 1 | 2 |
| 47 | 1 | 2 | 2 |
| 5 | 2 | 2 | 2 |
| 31 | 3 | 2 | 2 |
| 17 | 4 | 2 | 2 |
| 4 | 5 | 2 | 2 |
| 12 | 6 | 2 | 2 |
| 59 | 7 | 2 | 2 |
| 31 | 8 | 2 | 2 |
| 43 | 9 | 2 | 2 |
| 85 | 10 | 2 | 2 |
| 0 | 1 | 3 | 2 |
| 14 | 2 | 3 | 2 |
| 0 | 3 | 3 | 2 |
| 0 | 4 | 3 | 2 |
| 0 | 5 | 3 | 2 |
| 1 | 6 | 3 | 2 |
| 0 | 7 | 3 | 2 |
| 0 | 8 | 3 | 2 |
| 1 | 9 | 3 | 2 |
| 0 | 10 | 3 | 2 |
| 31 | 1 | 1 | 3 |
| 17 | 2 | 1 | 3 |
| 28 | 3 | 1 | 3 |
| 18 | 4 | 1 | 3 |
| 17 | 5 | 1 | 3 |
| 18 | 6 | 1 | 3 |
| 15 | 7 | 1 | 3 |
| 13 | 8 | 1 | 3 |
| 24 | 9 | 1 | 3 |
| 24 | 10 | 1 | 3 |
| 24 | 1 | 2 | 3 |
| 17 | 2 | 2 | 3 |
| 13 | 3 | 2 | 3 |
| 22 | 4 | 2 | 3 |
| 15 | 5 | 2 | 3 |
| 22 | 6 | 2 | 3 |
| 14 | 7 | 2 | 3 |
| 21 | 8 | 2 | 3 |
| 14 | 9 | 2 | 3 |
| 10 | 10 | 2 | 3 |
| 9 | 1 | 3 | 3 |
| 15 | 2 | 3 | 3 |
| 15 | 3 | 3 | 3 |
| 11 | 4 | 3 | 3 |
| 14 | 5 | 3 | 3 |
| 18 | 6 | 3 | 3 |
| 9 | 7 | 3 | 3 |
| 9 | 8 | 3 | 3 |
| 14 | 9 | 3 | 3 |
| 15 | 10 | 3 | 3 |
| 13 | 1 | 1 | 4 |
| 3 | 2 | 1 | 4 |
| 19 | 3 | 1 | 4 |
| 16 | 4 | 1 | 4 |
| 23 | 5 | 1 | 4 |
| 21 | 6 | 1 | 4 |
| 13 | 7 | 1 | 4 |
| 12 | 8 | 1 | 4 |
| 6 | 9 | 1 | 4 |
| 8 | 10 | 1 | 4 |
| 13 | 1 | 2 | 4 |
| 19 | 2 | 2 | 4 |
| 9 | 3 | 2 | 4 |
| 6 | 4 | 2 | 4 |
| 9 | 5 | 2 | 4 |
| 14 | 6 | 2 | 4 |
| 12 | 7 | 2 | 4 |
| 2 | 8 | 2 | 4 |
| 9 | 9 | 2 | 4 |
| 24 | 10 | 2 | 4 |
| 12 | 1 | 3 | 4 |
| 8 | 2 | 3 | 4 |
| 9 | 3 | 3 | 4 |
| 7 | 4 | 3 | 4 |
| 7 | 5 | 3 | 4 |
| 10 | 6 | 3 | 4 |
| 6 | 7 | 3 | 4 |
| 10 | 8 | 3 | 4 |
| 18 | 9 | 3 | 4 |
| 5 | 10 | 3 | 4 |
